# Ziprasidone-Induced Oculogyric Crisis in a 74-Year-Old Female

**DOI:** 10.7759/cureus.9100

**Published:** 2020-07-09

**Authors:** Alphonso Wilson, Asia Filatov, Mishah Azhar, Marc Swerdloff, Sameea Husain Wilson

**Affiliations:** 1 Internal Medicine, Charles E. Schmidt College of Medicine, Florida Atlantic University, Boca Raton, USA; 2 Neurology, Charles E. Schmidt College of Medicine, Florida Atlantic University, Boca Raton, USA; 3 Neurology, Marcus Neuroscience Institute, Boca Raton, USA

**Keywords:** ziprasidone, oculogyric crisis, acute dystonia

## Abstract

Oculogyric crisis is a rare ocular dystonia first appearing at the turn of the last century in postencephalitic patients. In the modern era, they were most frequently associated with first-generation D1 dopaminergic receptor blocking antipsychotic medication.

We present an unusual case of acute oculogyric crisis in a 74-year-old woman with long-standing Parkinson disease following exposure to the second-generation neuroleptic ziprasidone, which has dopaminergic (D2) and serotoninergic (5-HT2A) receptor blocking effects and is used for severe delusions and psychosis. To the best of our knowledge, there are no other published reports.

## Introduction

Acute motor adverse events are common with the use of first-generation typical anti-dopaminergic medications prompting the development of atypical second-generation agents [1]. These motor disorders are divided into akathisias (10%-40%), acute dystonias (2%-3%) and neuroleptic malignant syndrome (0.2%). Acute dystonias are motor disorders characterized by uncontrollable muscular contractions usually occurring within days of exposure to the inciting medication typically restricted to the musculature of the head, pharynx, cervical spine and trunk [1]. 

Oculogyric crisis (OGC) is the rarest of the acute dystonias presenting as involuntary painless contractions of the extraocular muscles resulting in sustained upward eye deviation [2]. These episodes may last for minutes to hours. Their mechanism of action is unknown but possibly due to acute selective dopaminergic blockade [3].

We report a case of acute OGC in a hospitalized 74-year-old woman approximately one month following outpatient administration of ziprasidone. Ziprasidone is a commonly prescribed second-generation atypical neuroleptic with antiserotonin and antidopaminergic effects. There are four previously reported cases of ziprasidone-associated OGC in patients treated for schizophrenia [4-7]. This is the first case of ziprasidone-induced OGC in a patient with advanced Parkinson disease.

## Case presentation

Our patient is a 74-year-old woman with a past medical history of long-standing Parkinson disease, diabetes mellitus and hypothyroidism who presented to the emergency department with generalized weakness and episodic confusion. She complained of troubling sialorrhea, dysphagia, random eye movement behavioral sleep disorder and visual hallucinations. For three days, she complained of frequent spasm of her eyelids during this time among other somatic concerns. She denied pain with eye movement but had no self-awareness as to whether she was in control of her eyes and eyelid spasms. She was prescribed pimavanserin for psychosis and rivastigmine for memory loss.

Her current medications at the time included carbidopa/levodopa, liothyronine, rivastigmine, clonazepam, levothyroxine and pimavanserin. Our initial diagnosis was toxic encephalopathy in the setting of her Parkinson disease and dementia. On physical examination, she was alert and able to follow commands when not in crisis. On neurologic examination, she exhibited hypomimia with marked four limb rigidity and bradykinesia. During the crisis there was intermittent sustained upward deviation noted of the eyes compounded by a decrease in mentation (Video 1). When the crisis was over, her eye movements were intact. There was no associated neck deviation or tongue protrusion. 

**Video 1:** Intermittent sustained upward deviation of the eyes seen on physical exam.

**Figure 1:**
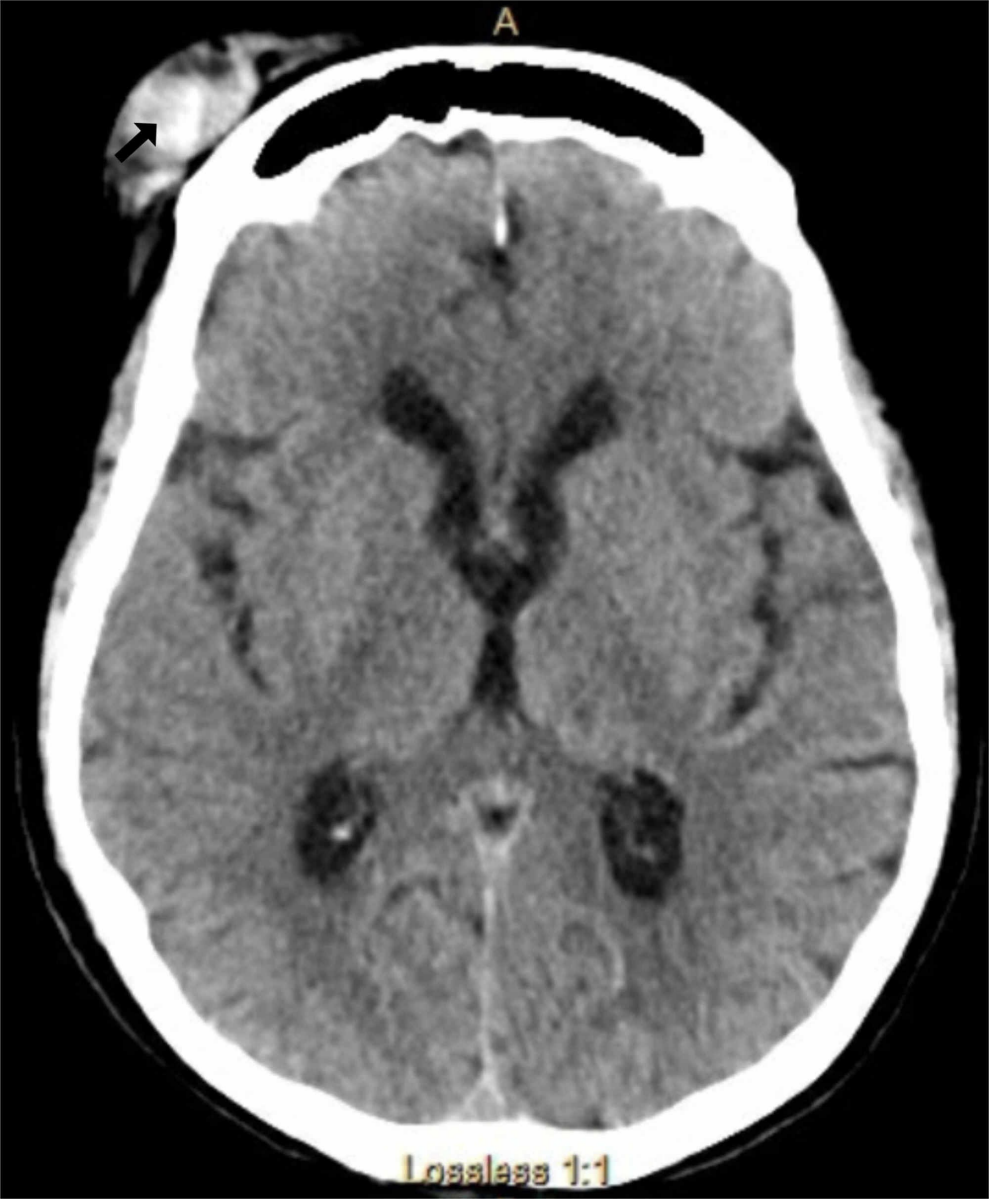
A CT image of the head demonstrates an extra-axial hematoma overlying the frontal bone. This finding is not relevant to the case report, as a hematoma cannot cause an oculogyric crisis.

Lab work including complete blood count, urinalysis, blood culture and a chest x-ray did not reveal infection. A CT image of the head demonstrated an extra-axial hematoma from the left frontal bone (Figure 1).

**Figure 2:**
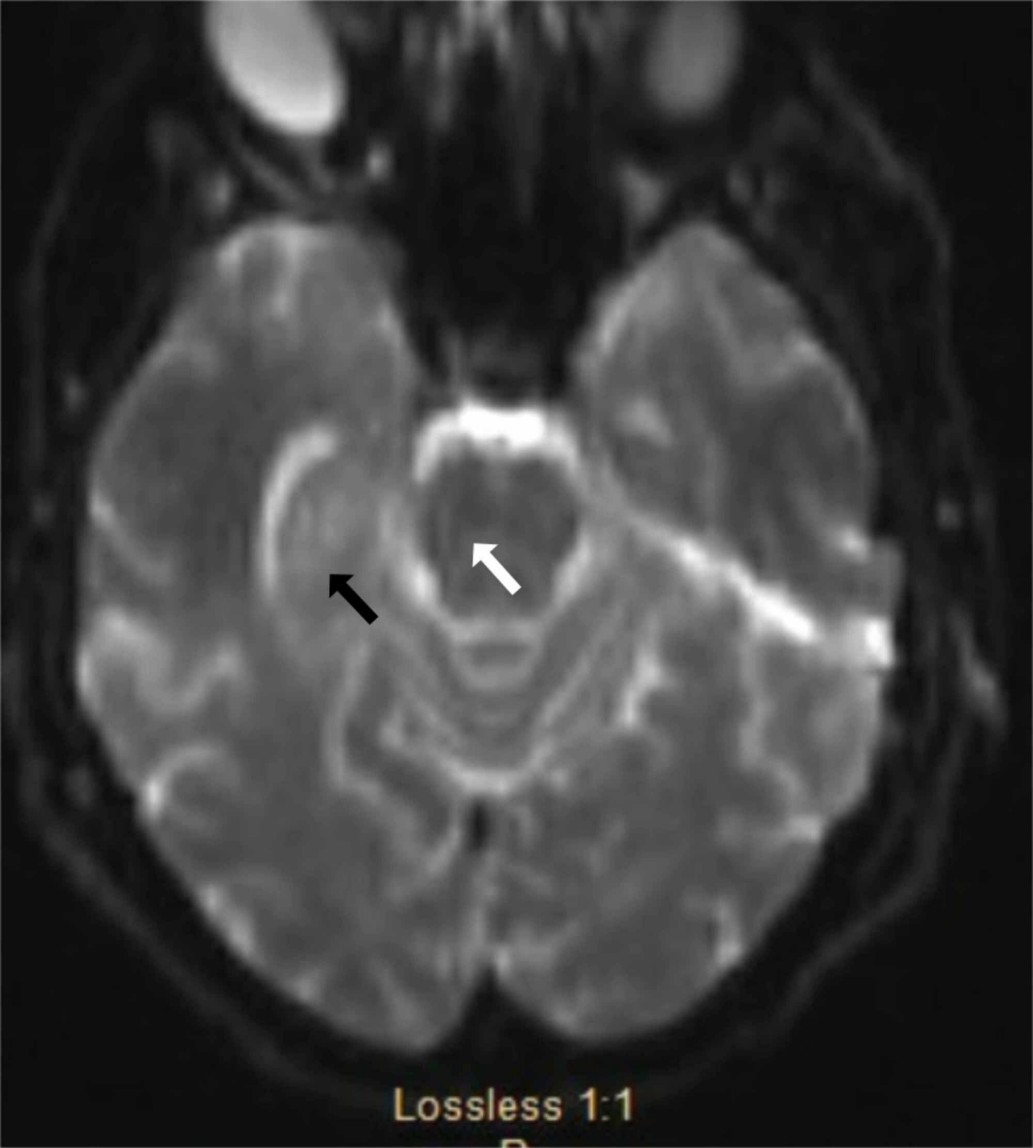
MRI of the brain demonstrates small vessel disease (black arrow) and a right parietal and pontine cavernoma (white arrow).

This was thought to be a potential seizure focus and the etiology of her encephalopathy. Her marked rigidity and altered mental status raised concerns of neuroleptic malignant syndrome but lack of fever opposed this hypothesis. MRI of the brain showed small vessel disease and a right parietal and pontine cavernoma (Figure 2).

**Figure 3:**
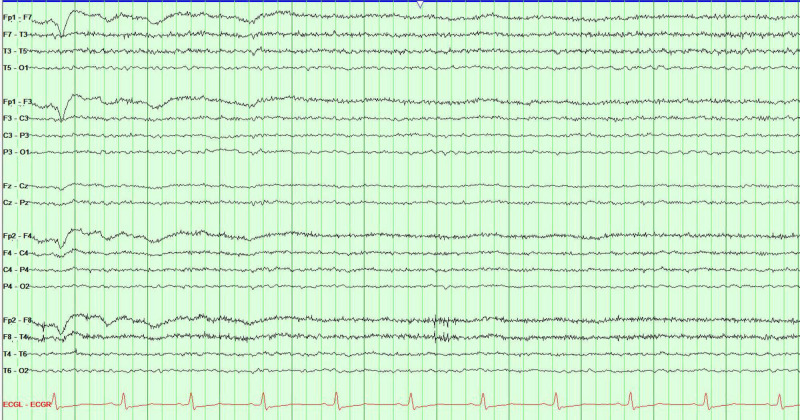
Electroencephalogram without epileptiform activity.

Electroencephalogram was negative for epileptiform activity (Figure 3). Her husband revealed she was recently treated for severe delusions and psychosis with the atypical antidopaminergic agent ziprasidone within the last month. 

She was diagnosed with OGC secondary to exposure to a second-generation antipsychotic. First- and second-line treatments, namely benztropine, diphenhydramine and benzodiazepines, were not prescribed for fear of depressing an already compromised mental state. Her carbidopa/levodopa dosage was subsequently increased to 1 tab po QID. Following intervention her oculogyric symptoms were reduced, and the patient was eventually discharged.

## Discussion

OGC is a rare dystonia recognized as sustained upward ocular deviations that are conjugate, tonic and/or paroxysmal [3]. The condition often occurs after administration of typical antipsychotics, antiepileptics, antiemetics, antimalarials or antidepressants. The patient may exhibit bilateral dystonic elevation of visual gaze that may last for a prolonged period. An accurate history is essential to choose between other causes of ocular deviation [8]. 

Although this condition is more frequent in children, it may be seen in elderly patients. It occurs after an imbalance in the nigrostriatal dopamine/cholinergic tone. Dopamine D2 receptor blockade leads to an excess of striatal cholinergic output. This is usually dose independent and may occur at therapeutic dosages [3]. Generally, men are more frequently affected [9]. A family history of neurological disease and remote treatment with neuroleptics do put patients at increased risk of OGC [10]. 

The pathophysiology of OGC remains elusive given our patient’s polypharmacy [11]. Her longstanding disease of the nigrostriatal dopaminergic pathway with its functional disruption of dopaminergic neurotransmission predisposed her to the development of OGC as has been seen with postencephalitic parkinsonism cases [12].

Treatment of OGCs depends on the etiology of the condition [13]. In our case, the initial step required the removal of the offending agent or the reduction of its dosage. In cases where this is not possible, administration of anticholinergics, such as benztropine or antihistamines, is used [14]. Repeated administration after 15 to 30 minutes may be needed [15].

## Conclusions

OGC is presently infrequently encountered in the general patient population. Second-generation antidopaminergic medications are thought to have less adverse extrapyramidal side effects as compared to first-generation medications. It was surprising for us to see OGC caused by a medication with low risk for this adverse event. Ziprasidone-induced OGC is an atypical phenomenon. It is unknown what role the novel antipsychotic pimavanserin has to play in the resurgence of OGC with and without exposure to a second-generation antipsychotic.

## References

[1] Dressler D, Benecke R (2005). Diagnosis and management of acute movement disorders. J Neurol.

[2] Richa S, Yazbek J-C (2010). Ocular adverse effects of common psychotropic agents. CNS Drugs.

[3] Slow EJ, Lang AE (2017). Oculogyric crises: a review of phenomenology, etiology, pathogenesis, and treatment. Mov Disord.

[4] Ramos AE, Shytle RD, Silver AA, Sanberg PR (2003). Ziprasidone-induced oculogyric crisis. J Am Acad Child Adolesc Psychiatry.

[5] Rosenfield PJ, Girgis RR, Gil R (2007). High-dose ziprasidone-induced acute dystonia. Prog Neuropsychopharmacol Biol Psychiatry.

[6] Gupta S, Nolan TN, Frank BL (2008). Case report of oculogyric crisis with ziprasidone in a minor. Prim Care Companion J Clin Psychiatry.

[7] Viana Bde M, Prais HA, Camargos ST, Cardoso FE (2009). Ziprasidone-related oculogyric crisis in an adult. Clin Neurol Neurosurg.

[8] Tokgöz Y, Öznur T, Bolu A, Cemil C, Özcan U (2018). Tardive oculogyric crisis during treatment with amisulpride. Klinik Psikofarmakoloji Bulteni.

[9] van Harten PN, Hoek HW, Kahn RS (1999). Acute dystonia induced by drug treatment. BMJ.

[10] Nebhinani N, Suthar N (2017). Oculogyric crisis with atypical antipsychotics: a case series. Indian J Psychiatry.

[11] Solberg M, Koht J (2017). Oculogyric crises. Tremor Other Hyperkinet Mov.

[12] Barow E, Schneider SA, Bhatia KP, Ganos C (2017). Oculogyric crises: etiology, pathophysiology and therapeutic approaches. Parkinsonism Relat Disord.

[13] Gold DR (2019). Eye movement disorders: conjugate gaze abnormalities. Liu, Volpe, and Galetta's Neuro-Ophthalmology.

[14] Cheng KJ, Yeh TC, Ku SC, Tseng YT, Liang CS (2020). Electroconvulsive therapy for anticholinergic and benzodiazepine nonresponsive tardive oculogyric crisis: a case report. Am J Ther.

[15] Kim M, Lee D, Lee D, Koh I, Ehm G (2019). Oculogyric crisis following single administration of clebopride maleate. Neurology Asia.

